# Assessment of Mental Foramen Characteristics in Syrian Population Using Cone Beam Computed Tomography: A Pilot Study

**DOI:** 10.7759/cureus.58549

**Published:** 2024-04-18

**Authors:** Radwan A Haffaf, Mohamad M Younes, Ali Y Shqera, Mounzer Assad, Abdul-Kareem Hasan

**Affiliations:** 1 Department of Orthodontics, Tishreen University, Latakia, SYR; 2 Orthodontics, Ajyad Medical Center, Sharjah, ARE; 3 Department of Oral and Maxillofacial Surgery, Manara University, Latakia, SYR; 4 Department of Oral and Maxillofacial Surgery, Tishreen University, Latakia, SYR

**Keywords:** oral medicine, dentistry, pilot study, mental foramen, cone beam computed tomography

## Abstract

Background

Knowledge of the mental foramen (MF) characteristics is crucial for avoiding iatrogenic injuries during dental implant placement, root canal treatment, orthognathic surgery, and other dental and surgical interventions. Cone beam computed tomography (CBCT) offers a valuable tool for evaluating the MF characteristics with its precise anatomical details. The current study investigates the horizontal and vertical position variations in addition to the exit angle of MF within the Syrian adult population.

Materials and methods

The sample included CBCT scans of 42 subjects with an equal number of males and females (21 males, 21 females), with no underlying pathology in the investigated region, mean age was 24.7 years (SD: 7.2 years). CBCT scans were retrospectively analyzed in terms of the vertical, horizontal, and exit angle direction of MF. The chi-square test was conducted to investigate statistical differences in terms of MF horizontal and vertical positions. A T-test was conducted to investigate statistical differences in terms of exit angle direction. Comparisons were conducted between males and females groups, and between the left and right sides in the total sample group.

Results

The most frequent horizontal position was position 3 (MF between the first and second premolars) on the right side (n=20, 47.61%), and on the left side (n=21, 50%). The most frequent vertical position was position 3 (MF below the apices of the premolars) on the right side (n=29, 69.04%), and on the left side (n=27, 64.28%). The exit angle of the MF was in a backward direction, with a mean value of 118.42° (SD: 6.45 degrees), and 115.97° (SD: 7.29 degrees) on the right and left side, respectively. Statistically significant differences were found in terms of the right vertical position between males and females (P value < 0.05).

Conclusion

Variations in MF characteristics exist in the Syrian population. Statistically significant differences were found in the right vertical position of MF. The current study findings necessitate precise preoperative three-dimensional imaging for dental interventions among this population. By establishing normative values for the Syrian population, the results can contribute to improved surgical planning and patient care, and can be used for comparative studies for more understanding of the human anatomical variations.

## Introduction

The mental foramen (MF) is a bilateral anatomical aperture situated on the anterolateral aspect of the mandible. It includes a neurovascular complex that provides both sensory innervation and vascular supply to the perioral region, including the angle of the mouth, the lower lip, and the labial mucoperiosteum of the anterior mandibular arch [1,2].

Intravascular injection of a local anesthetic during mental nerve blocks can lead to serious complications, such as toxicity, hematoma formation, mental nerve injury, and failed nerve block. These complications are of particular concern to emergency department practitioners, as they may be less familiar with the relevant anatomy [2,3].

The precise identification of the MF has been the subject of numerous investigations, encompassing its anatomical location, radiographic size, and morphology. This accurate localization is imperative for both diagnostic purposes and routine clinical practice [3,4].

Orthodontic mini-implant insertion, endodontic procedures concerning premolars, parasymphyseal fractures, osteotomy procedures during orthognathic surgery, implant dentistry, and removable prosthodontic interventions significantly influence the anatomical characterization and positioning of the MF [3]. The influence of ethnicity on specific dental characteristics has been well-documented in scientific literature [1,2]. Accurate clinical or radiographic identification of MF is a prerequisite for various routine dental interventions [3].

Multiple methodologies have been employed to delineate the characteristics and anatomical location of the MF, including panoramic radiography, ultrasound, dry skull examination, computed tomography (CT), and cone beam computed tomography (CBCT). These techniques provide complementary insights into the MF position, dimensions, and relationship to adjacent anatomical structures [1-4].

The anatomical location of the MF is subject to individual variability, influenced by factors such as sex, age, and ethnicity [3]. Despite the significance of this anatomical landmark for surgical procedures, limited information is available regarding MF characteristics in the Syrian population. This study aims to determine the approximate horizontal and vertical anatomical position and exit angle of the MF in Syrian individuals using CBCT.

## Materials and methods

This preliminary investigation utilized CBCT scans of 42 subjects with an equal number of males (n=21, 50%) and females (n=21, 50%). The participants ages ranged from 18 to 32 years with a mean age of 24.7 years (SD: 7.2 years). The material was retrospectively retrieved from the archives of the Departments of Orthodontics and Oral Surgery at the Faculty of Dentistry, Tishreen University, Latakia, Syrian Arab Republic. The study was approved by the Institutional Review Board of Tishreen University with the number 1108\2021. Informed consent had been obtained prior to data dissemination for scientific inquiry and publication. It is important to note that these scans were not acquired specifically for the purpose of this research, but rather for various therapeutic and diagnostic procedures, including orthodontic interventions, implant placement, endodontic treatments, and microsurgical interventions.

Inclusion criteria included: age between 18 and 32 years; CBCT scans covering the desired anatomical region; no history of trauma; and absence of teeth\bone anomalies in the region of interest. The following criteria were used to exclude potential participants: prior orthognathic or orthodontic treatment involving the extraction of lower premolars; the presence of periapical lesions within the region of interest; and any CBCT distortion attributable to metallic artifacts.

**Figure 1:**
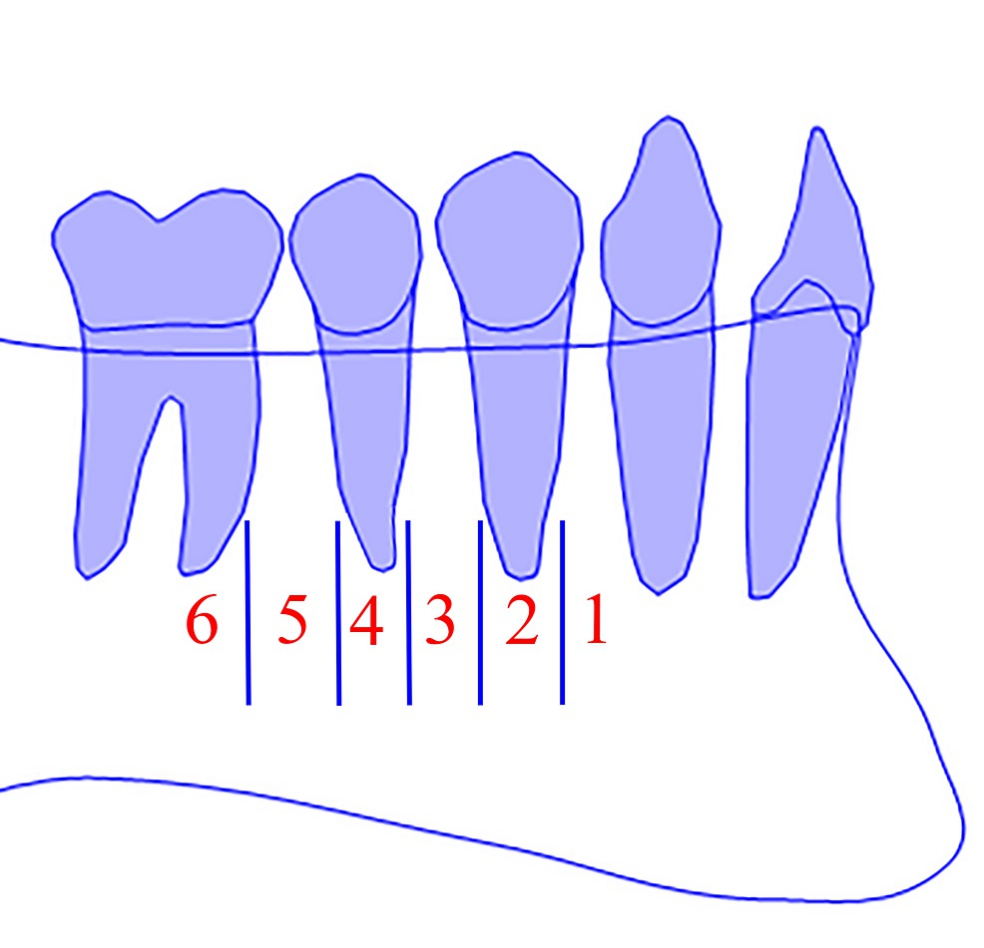
The mental foramen (MF) horizontal position classification (right side). Diagrammatic representation source: the authors.

The characteristics under investigation were the horizontal and vertical positions and the direction of the exit angle. These measurements were identified as follows:

The horizontal position was determined according to Tebo and Telford classification (Figure 1) [5]. The six horizontal categories were: (1) MF was between the canine and the first premolar, (2) MF was at the level of the first premolar, (3) MF was between the first and second premolars, (4) MF was at the level of the second premolar, (5) MF was between the second premolar and the first molar, and (6) MF was at the level of the first molar.

**Figure 2:**
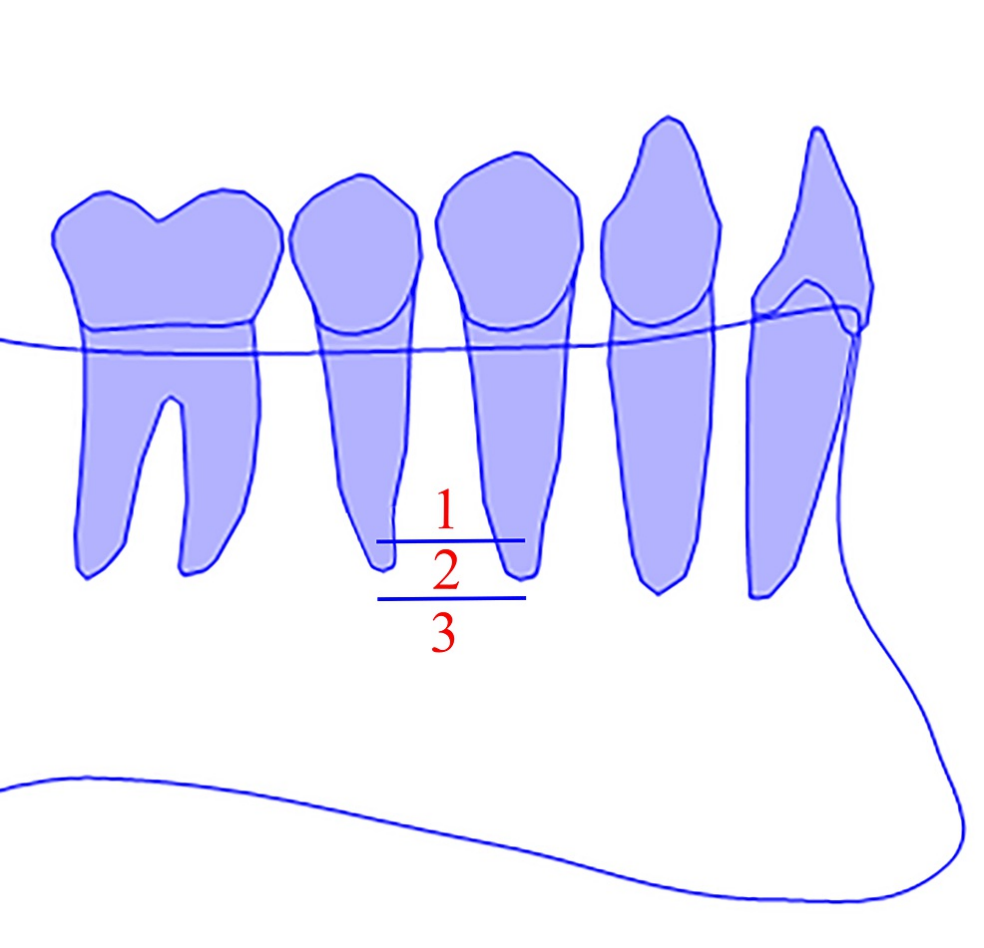
The mental foramen (MF) vertical position classification (right side). Diagrammatic representation source: the authors

The vertical position was determined using the same classification used by Zmyslowska-Polakowska et al. (Figure 2) [2]. The three vertical categories were: (1) MF was located above the level of the apices of the first and second mandibular premolar teeth, (2) MF was located at the level of the apices of the first and second mandibular premolar teeth, and (3) MF was located below the level of the apices of the first and second mandibular premolar teeth. Vertical and horizontal positions were determined on the sagittal plane.

**Figure 3:**
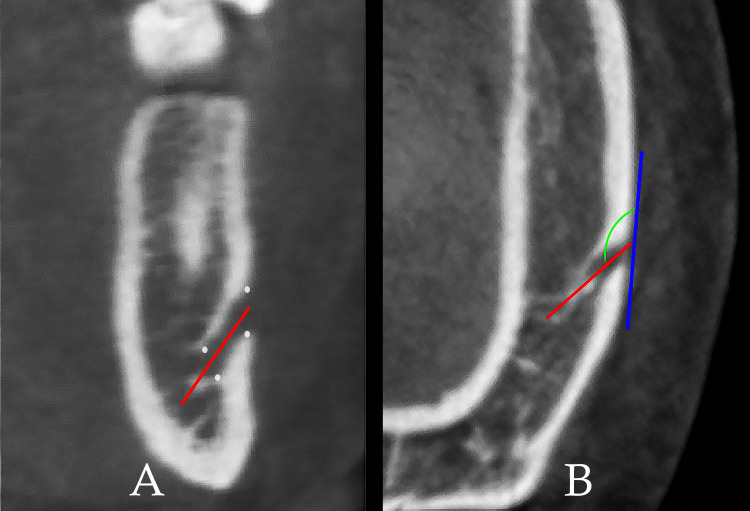
The exit angle is the angle formed by the mental foramen (MF) axis and a tangent to the external mandibular cortex. The exit angle (green line); the MF axis (red line); the external mandibular cortex (blue line). A: coronal plane. B: axial plane.

The exit angle was defined as the angle formed by the axis of the MF and a line tangential to the external mandibular cortex at the opening of the MF. The canal axes were identified on the coronal plane (Figure 3A). The outer tangent and the exit angle were identified and measured on the axial plane (Figure 3B).

The exit angle direction was categorized into three groups. Angles greater than 90 degrees were classified as backward, angles equal to 90 degrees were considered perpendicular, and angles less than 90 degrees were designated as forward.

All CBCT images were analyzed using OnDemand 3D App 1.0.10.5385 (Cybermed Inc., Seoul, South Korea). The software was installed on the Windows 7 64-bit operating system (Microsoft, Redmond, WA, USA). All images were initially re-oriented using the Frankfort plane-based method (four landmarks), which was a feature included in the software. The used landmarks were: N (Nasion), L Or (Left Orbitale), R Or (Right Orbitale), and R Po (Right Porion).

Statistical analysis

Statistical Package for the Social Sciences (SPSS) version 20 (IBM Corp., Armonk, NY, USA) was used for statistical analysis. The chi-square test was utilized to assess the statistical differences in terms of the horizontal and vertical positions. The T-test was utilized to assess the statistical differences in terms of the exit angle. Statistical significance was assessed at p < 0.05. Descriptive statistics were calculated for the entire sample and for the subgroups.

## Results

The horizontal position 3, defined as the MF situated between the first and second premolars, exhibited a predominant frequency of 47.61% (n=20) and 50% (n=21) on the right and left sides, respectively. However, horizontal position 4, characterized by the MF positioned at the level of the second premolar, emerged as the second most prevalent position within the overall sample. This position was observed with frequencies of 40.47% (n=17) and 35.71% (n=15) on the right and left sides, respectively. Table 1 presents the statistical data and variations in horizontal position between the left and right sides within the overall sample. It is noteworthy that in three instances (7.14%), MF was identified in a horizontal position intermediate between the second premolar and the first molar on the left side. Additionally, one case (2.38%) of this positional variation was observed on the right side. However, no statistically significant differences were found between the right and left sides (P value > 0.05).

**Table 1:** Total sample Horizontal position statistics. Data represented as frequency count (n) and percentage (%). P value < 0.05 was considered significant.

-	Right horizontal position			Left horizontal position		
-	Frequency (n)	Percentage (%)	Cumulative Percentage (%)	Frequency (n)	Percentage (%)	Cumulative Percentage (%)
1	0	0	0	0	0	0
2	4	9.52	9.52	3	7.14	7.14
3	20	47.61	57.13	21	50.00	57.14
4	17	40.47	97.60	15	35.71	92.85
5	1	2.38	100.00	3	7.14	100.00
Total	42	100.00	-	42	100.00	-
Chi-square - P value (0.72)						

The most frequent vertical position was position 3 (MF below the apices of the premolars), with a frequency rate of 69% (n=29) on the right side and 64.28% (n=27) on the left side. Table 2 presents the statistical data and variations in vertical position between the left and right sides within the overall sample. No statistically significant differences were found (P value > 0.05).

**Table 2:** Total sample vertical position statistics. Data represented as frequency count (n) and percentage (%). P value < 0.05 was considered significant.

-	Right vertical position			Left vertical position		
-	Frequency (n)	Percentage (%)	Cumulative Percentage (%)	Frequency (n)	Percentage (%)	Cumulative Percentage (%)
1	2	4.76	4.76	1	2.38	2.38
2	11	26.19	30.95	14	33.33	35.71
3	29	69.04	100.00	27	64.28	100.00
Total	42	100.00	-	42	100.00	-
Chi-square - P value (0.68)						

Within the overall sample population, the MF exhibited a posterior exit angle. Table 3 presents the statistical data and variations in exit angle measurements for both left and right sides in the entirety of the sample. Statistical analysis revealed no significant differences in the exit angle between the right and left sides (P value > 0.05).

**Table 3:** Total sample exit angle statistics. Data represented as frequency count (n) and degrees (°). P value < 0.05 was considered significant. SD: standard deviation.

-		Right angle	Left angle
Frequency (n)	Valid	42	42
	Missing	0	0
Mean (°)		118.42	115.97
Standard Error of Mean (°)		0.99	1.125
Median (°)		117.26	115.85
SD (°)		6.45	7.29
Range (°)		28.12	34.63
Minimum (°)		102.88	92.97
Maximum (°)		131.00	127.60
Percentiles	25	113.82	111.58
	50	117.26	115.85
	75	123.28	122.20
T-test - P value (0.10)			

Statistical analysis of the exit angle, horizontal position, and vertical position yielded no other significant differences within the total study population (p > 0.05), as evidenced in Tables 1-3. However, when examining the female and male subgroups separately, a statistically significant difference was found in the vertical position on the right side (p = 0.015), as indicated in Tables 4-6. Tables 4-6 present the outcomes of statistical comparisons between females and males in terms of the vertical and horizontal positions, as well as the exit angle.

**Table 4:** Horizontal position comparison between females and males. Data represented as frequency count (n) and percentage (%). P value < 0.05 was considered significant.

-	Right horizontal position				Left horizontal position			
-	Frequency (n)		Percentage (%)		Frequency (n)		Percentage (%)	
-	Female	Male	Female	Male	Female	Male	Female	Male
1	0	0	0	0	0	0	0	0
2	2	2	9.52	9.52	0	3	0	14.28
3	10	10	47.61	47.61	12	9	57.14	42.85
4	8	9	38.09	42.85	7	8	33.33	38.09
5	1	0	4.76	0	2	1	9.52	4.76
Total	21	21	100.00	100.00	21	21	100.00	100.00
-	Chi-square - P value (0.69)				Chi-square - P value (0.17)			

**Table 5:** Vertical position comparison between females and males. Data represented as frequency count (n) and percentage (%). P value < 0.05 was considered significant. * Statistically significant.

-	Right vertical position				Left vertical position			
-	Frequency (n)		Percentage (%)		Frequency (n)		Percentage (%)	
-	Female	Male	Female	Male	Female	Male	Female	Male
1	2	0	9.52	0	1	0	4.76	0
2	2	9	9.52	42.85	8	6	38.09	28.57
3	17	12	80.95	57.14	12	15	57.14	71.42
Total	21	21	100.00	100.00	21	21	100.00	100.00
-	Chi-square - P value (0.01)*				Chi-square - P value (0.36)			

**Table 6:** Exit angle comparison between females and males. Data represented as frequency count (n) and degrees (°). P value < 0.05 was considered significant. SD: standard deviation.

-		Right angle		Left angle	
-	-	Female	Male	Female	Male
Frequency (n)	Valid	21	21	21	21
	Missing	0	0	0	0
Mean (°)		118.15	118.70	115.48	116.46
Standard. Error of Mean (°)		1.54	1.29	1.810	1.37
Median (°)		117.69	117.12	115.79	117.38
SD (°)		7.10	5.91	8.29	6.30
Range (°)		28.12	22.13	34.55	26.94
Minimum (°)		102.88	107.70	92.97	100.66
Maximum (°)		131.00	129.83	127.52	127.60
Percentiles	25	112.28	115.64	110.58	111.78
	50	117.69	117.12	115.79	117.38
	75	123.15	123.40	122.86	121.37
-		T-test - P value (0.77)		T-test - P value (0.67)	

## Discussion

The present investigation aimed to ascertain the anatomical characteristics of the MF in the adult Syrian population. The MF positioning in both horizontal and vertical planes was evaluated. Furthermore, the angle of emergence at the MF orifice was quantified. The acquisition of normative values specific to the Syrian population serves as a valuable tool for optimizing surgical planning and enhancing patient outcomes. One recognized limitation of this study is its retrospective nature. Nevertheless, this limitation is ethically justifiable as it would have been unethical to subject individuals to additional radiation exposure solely for the purposes of anatomical research. A larger population size would also lead to generalizable results and reflect the original population characteristics more precisely.

CBCT is a state-of-the-art imaging technique that provides high-resolution three-dimensional images of the maxillofacial region. This enables clinicians to evaluate the anatomical characteristics of maxillofacial structures with greater precision [6]. CBCT offers comprehensive information regarding the location and orientation of the MF relative to adjacent anatomical structures. Additionally, it allows for accurate determination of the MF exit angle. These precise measurements reduce the risk of iatrogenic nerve damage during surgical interventions. Moreover, CBCT facilitates the detection and assessment of variations in MF anatomy. Such variations may significantly impact surgical planning and clinical outcomes. The detailed anatomical information provided by CBCT enhances the accuracy and safety of dental procedures involving the MF [2,4,6].

The accurate characterization of MF characteristics is paramount in surgical procedures involving implant placement, nerve block administration, diagnosis, and treatment of pathological conditions affecting the inferior alveolar nerve [2,3,7]. As Saha and Nair have demonstrated, data concerning MF characteristics are indispensable for meticulous implant planning and can be readily obtained from CBCT imaging [8]. This facilitates the formulation of well-defined treatment strategies, thereby minimizing the potential for surgical complications [7,8].

The present study data demonstrated that the MF was located between the first and second premolars in nearly half of the sample. Statistical analysis revealed no significant difference (P value > 0.05) in MF horizontal positioning between the left and right sides or between male and female subjects. Notably, in 2.38% (n=1) and 7.14% (n=3) of the overall sample, the MF was atypically situated between the second premolar and the first permanent molar on the right and left sides, respectively. These observations align with the findings of a recent systematic review conducted by Pelé et al. [9]. Generally, it was reported in previous studies in the Arabic population that the MF was predominantly positioned anteroposteriorly at the level of the second premolar [10].

Previous studies have mentioned some anatomical variations like the presence of accessory MF [11]. However, addressing this type of anatomical variations was beyond the scope of the current preliminary study. Because of its low prevalence, these variations need further investigations with a larger sample size.

Mental nerve block injection, employed in dental anesthesia, targets the region adjacent to the roots of the mandibular premolars [7,12]. In the current study, statistical evidence suggests that the foramen exhibits a predilection for specific anatomical locations. Position 3, situated interproximally between mandibular premolars, manifests a prevalence of 47-50% (n=20 and 21 respectively), while Position 4, aligned with the second premolar, presents with a frequency of 35-40% (n=15-17). These aforementioned positions constitute the most prevalent sites for mental nerve block [12]. Consideration of these data is paramount in assessing the efficacy of mental nerve block relative to inferior alveolar nerve block in the Syrian population.

The results obtained in the current study correspond to those reported by Okiriamu et al. (2023) regarding the Kenyan population [13]. In more than half of their sample, the MF was situated anterior to the second premolars. Analysis revealed no statistically significant difference between males and females concerning this horizontal position. These findings align with those presented by Rath et al. pertaining to the Eastern Indian population [14].

MF was consistently located inferior to the apices of the mandibular premolars in the majority of cases (29 cases on the right side and 27 cases on the left side). Statistical analysis revealed no significant difference between the right and left sides within the overall sample (P value > 0.05). However, a statistically significant difference between males and females was observed specifically on the right side (P value < 0.05). These findings align with previous observations reported by Zmyslowska-Polakowska et al. among the Polish population [2]. Rath et al. reported a statistically significant difference between the right and left sides in females only [14]. Study design and sample size may have contributed to these discrepancies.

Within the overall sample, MF exit angle exhibited a posterior orientation, characterized by an average value of 118 degrees on the right side and 115 degrees on the left side. Statistical analysis revealed negligible differences between the angles observed in male and female subgroups (P value > 0.05). Furthermore, all subgroups exhibited a consistent pattern of the angle direction, with no statistically significant differences identified (P value > 0.05). These findings corroborate previous observations reported by Goyushov et al. [15].

A preliminary investigation conducted by Sheikhi et al. demonstrated that the predominant anatomical positioning of the MF was apically located to the apex of the mandible premolars and aligned with the second premolar tooth. Notably, the trajectory of the foramen exit point deviated posteriorly, as found in the present study [16].

Balcioglu et al.'s investigations offer compelling evidence that MF undergoes a consistent posterior displacement during mandibular development. Postnatally, the MF exhibits a regularized posterior migration pattern [17].

In addition to its clinical significance, the current study also contributes to the scientific understanding of human anatomy and variation. The collected data can be used for comparative studies and in the development of anatomical atlases and databases. It is the first study that investigates this topic in the Syrian population using CBCT scans, which may be used in further investigations in the fields of oral and maxillofacial surgery, dentistry, forensic dentistry and anatomy.

The study's limitations include the small sample size and the retrospective pilot study design, which was justified from an ethical viewpoint to avoid unnecessary patient radiation. Another limitation is the wide age range.

## Conclusions

The MF exhibits variations beyond the most prevalent types. These variations necessitate comprehensive preoperative imaging, particularly three-dimensional images, to precisely locate the foramen and guide dental and surgical interventions. However, the most frequent position of the MF was the horizontal position 3 (between the first and second premolars) and the vertical position 3 (below the apices of the premolars). The exit angle of the MF was in a backward direction.

The data obtained can contribute to enhanced preoperative planning and patient management. By establishing normative values specific to the Syrian population, the results can facilitate improved surgical planning and patient care, serving as a foundation for further comparative studies to deepen the understanding of human anatomical variations.
